# Public Health Workforce Burnout in the COVID-19 Response in the U.S.

**DOI:** 10.3390/ijerph18084369

**Published:** 2021-04-20

**Authors:** Kahler W. Stone, Kristina W. Kintziger, Meredith A. Jagger, Jennifer A. Horney

**Affiliations:** 1 Department of Health and Human Performance, Middle Tennessee State University, Murfreesboro, TN 37132, USA; kahler.stone@mtsu.edu; 2 Department of Public Health, University of Tennessee, Knoxville, TN 37996, USA; kkintzig@utk.edu; 3 Independent Researcher, Austin, TX 78704, USA; mph.jagger@use.startmail.com; 4 Epidemiology Program, University of Delaware, Newark, DE 19713, USA

**Keywords:** COVID-19, public health, workforce, burnout, pandemic response

## Abstract

While the health impacts of the COVID-19 pandemic on frontline health care workers have been well described, the effects of the COVID-19 response on the U.S. public health workforce, which has been impacted by the prolonged public health response to the pandemic, has not been adequately characterized. A cross-sectional survey of public health professionals was conducted to assess mental and physical health, risk and protective factors for burnout, and short- and long-term career decisions during the pandemic response. The survey was completed online using the Qualtrics survey platform. Descriptive statistics and prevalence ratios (95% confidence intervals) were calculated. Among responses received from 23 August and 11 September 2020, 66.2% of public health workers reported burnout. Those with more work experience (1–4 vs. <1 years: prevalence ratio (PR) = 1.90, 95% confidence interval (CI) = 1.08−3.36; 5–9 vs. <1 years: PR = 1.89, CI = 1.07−3.34) or working in academic settings (vs. practice: PR = 1.31, CI = 1.08–1.58) were most likely to report burnout. As of September 2020, 23.6% fewer respondents planned to remain in the U.S. public health workforce for three or more years compared to their retrospectively reported January 2020 plans. A large-scale public health emergency response places unsustainable burdens on an already underfunded and understaffed public health workforce. Pandemic-related burnout threatens the U.S. public health workforce’s future when many challenges related to the ongoing COVID-19 response remain unaddressed.

## 1. Introduction

The COVID-19 pandemic has affected millions across the world. As of the first week of February 2021, there were nearly 106 million confirmed cases and 2.3 million deaths globally [1]. In the U.S., the first confirmed case was documented on 20 January 2020 [2], and in the first eight months, the number of cases has grown to over 27 million, with an observed case–fatality ratio of 1.8% [3].

Public health emergencies such as COVID-19 not only affect those infected with the disease, they profoundly impact the frontline workforce tasked with patient care and emergency response. COVID-19 has had substantial impacts on the mental and physical health of frontline, patient-facing health care workers [4,5,6,7,8,9,10,11]. Multiple systematic reviews have found that health care workers are at an increased risk of developing mental health conditions such as psychological distress, insomnia, anxiety, depression, and symptoms of post-traumatic stress disorder [8,9,10]. In a survey of 1257 health care workers treating COVID-19 patients in China, 72% reported feeling distress, 34% reported experiencing insomnia, 50% reported symptoms of depression, and 45% reported symptoms of anxiety [7]. In terms of physical health, a review of 10 studies found that the most common physical health symptoms reported by health care workers were fever (85%), cough (70%), weakness (70%), as well as skin damage (97%) from prolonged use of personal protective equipment [12].

The detrimental mental and physical health effects on frontline workers will potentially diminish the size of the workforce due to burnout. In a study of 2040 hospital workers in Hong Kong during the severe acute respiratory syndrome (SARS) outbreak, frontline health care workers had higher self-reported anxiety, worry, and burnout compared to controls. Additionally, anxiety was correlated with more reported burnout [13]. Similar results were seen in a sample of Canadian health care workers. When compared to workers not treating individuals infected with SARS, those treating SARS patients reported higher levels of burnout, psychological distress, and post-traumatic stress. Further, one to two years after the SARS outbreak, 30% of those same Canadian health care workers still reported high burnout scores [14].

While the physical health, mental health, and burnout risk has been studied in frontline health care workers, to date, no study has examined the effects of COVID-19 on the public health workforce in the U.S. This may be, in part, due to the complexities of defining the U.S. public health workforce, which includes not only those who work for federal, state, and local governmental public health agencies (approximately 97,000 state and 147,000 local public health staff [15], but also workers in community based organizations, public health staff in health care systems, those working in academic public health, and others [16]. Defined more broadly, the U.S. public health workforce may include anyone engaged in activities that “assure the conditions within which people can be healthy,” which may include many workers across health care, environmental health, and health and safety [17]. The U.S. public health workforce also includes workers with various backgrounds working in diverse settings and roles such as physicians and nurses, managers, economists, and community development [18]. For this study, we defined the public health workforce as anyone with either an academic degree in a field related to public health or a professional role in a governmental or academic public health department that included participating in the public health response to COVID-19.

As the composition of the public health workforce is difficult to define, and varies by jurisdictions within the U.S. as well as globally, defining the public health workforce and its complex roles has made it difficult to assess its status [18]. However, the potential impact of burnout among public health workers is alarming, particularly given the documented decline in the U.S. governmental public health workforce, which lost 20% of its workforce, or 34,000 jobs, since 2008, while 62% of local health departments had flat or reduced funding and average overall declines in spending averaged 10.3% [19,20,21].

The objective of this study was to assess the mental and physical health of the public health workforce in the U.S., to identify risk and protective factors for burnout, and to describe short- and long-term career decisions resulting from the pandemic and response.

## 2. Materials and Methods

A cross-sectional survey was developed based on published scales to measure burnout and other effects of COVID-19 on the U.S. public health workforce using the web-based Qualtrics platform (Provo, UT, USA). The survey was pilot tested with public health staff at a large U.S. city health department and revised based on feedback. A link to the final survey was shared with members of professional networks, including the American Public Health Association’s Epidemiology Section and on social media with a group with documented training and workforce experience in public health (*n* = 3000). The results presented here represent responses during the first twenty days of data collection (23 August–11 September 2020).

The survey included 45 questions across three themes. Background characteristics included sociodemographic characteristics (age, gender, race/ethnicity, household characteristics, and education) and professional experience (years of experience, roles). Personal well-being included physical and mental health status (generalized anxiety, depression, physical and mental health days) and burnout among respondents. Career plans included asking participants their pre-pandemic vs. current plans (i.e., August/September 2020) to estimate trajectory changes due to the COVID-19 response.

Generalized anxiety disorder (GAD) was assessed using a previously validated 7-item (GAD-7) scale [22], and depressive disorder using the two-item Patient Health Questionnaire (PHQ-2) subscale [23]. We used a validated, non-proprietary single-item burnout measure to estimate self-reported burnout [24], categorized as some level of burnout (any symptoms of burnout: scale items 3–5) vs. none (items 1–2) and high level of burnout (items 4–5) vs. none/some (items 1–3). The number of poor physical health days, poor mental health days, and number of days of interrupted activity in the past 30 days questions were based on the Behavioral Risk Factor Surveillance System (BRFSS) 2019 questionnaire [25].

Data were downloaded from Qualtrics and analyzed using Stata Version 15 (College Station, TX, USA). Outcomes of interest (i.e., anxiety, depression, burnout, physical health days, and mental health days) were stratified by gender, age, race/ethnicity, marital status, household size, years of experience in public health, public health sector (i.e., practice, academic, other), and education. Geography was categorized using the U.S. Census Bureau regions [26]. To avoid overestimating prevalent outcomes with odds ratios, we reported prevalence ratios (PR) with 95% confidence intervals (CIs) calculated using Poisson regression with robust standard errors. Differences in career trajectories pre-pandemic to the current time were assessed with McNemar’s chi-square tests. The survey and related materials were reviewed by an Institutional Review Board and determined to be exempt.

## 3. Results

A total of 225 public health workers from 31 U.S. states and the District of Columbia completed the survey during the 20 day period. Over half (56.0%) of respondents were from the West Region, and the remaining were from the South (29.8%), Midwest (8.0%) and Northeast (6.2%). Most respondents were currently working in governmental public health agencies (78.9%). The majority were female (84.8%), White non-Hispanic (76.4%), and under the age of 40 (60.4%). This sample was more female than the overall U.S. public health workforce (84.8% compared to 77% female), more White (76.4% compared to 57.2%), and younger (mean age = 47) [15]. A diversity of public health roles (e.g., occupational health, mental health, vital statistics, injury, environmental health, public health emergency preparedness, informatics, substance abuse, maternal and child health, chronic disease, and infectious disease), years of public health experience and household characteristics were represented.

### 3.1. Mental and Physical Health of Public Health Workers

Symptoms of anxiety, depression, burnout, and poor physical health were widely reported (Table 1). Males reported more mental health disorders than females, including generalized anxiety (46.7% vs. 39.9%) and depression (33.3% vs. 28.2%), but reported fewer symptoms of burnout (60.0% vs. 68.3%). Respondents 50 years of age and older were less likely to report anxiety/depressive disorders and burnout than younger age groups. We noted few differences in anxiety or depressive disorders by race/ethnicity, though burnout was more likely to be reported by White, non-Hispanic individuals and other (including multiple) races (70.1% and 71.4%, respectively) than other groups. Individuals reporting other marital status (including widowed, divorced, separated, and other) reported fewer anxiety/depressive disorders and less burnout than individuals currently married or in a partnership or those who never married. Larger household sizes were associated with less anxiety or depressive disorders and less burnout. We did not note differences in anxiety or depressive disorders by years of experience in the U.S. public health workforce, public health sector, or education. However, more symptoms of burnout were reported by those with more than 15 years of experience (63.5% vs. <1-year experience: 38.1%), those in academia (85.2% vs. public health practice: 65.1%), and those with more education [master’s (67.2%) or doctoral degree (72.1%) vs. bachelor’s degree: 51.5%).

The proportion of respondents reporting poor health at least 14 out of the last 30 days was 13.6% for physical health (mean = 5.0 days), 41.4% for mental health (mean = 12.4 days), and 19.7% for reduced activity due to poor physical or mental health (mean = 6.7 days). Male respondents reported more poor mental health days than female respondents (13.7 vs. 12.1 days); however, females reported more poor physical health days (5.1 vs. 4.8 days) and more days where activities were impacted due to poor health (6.7 vs. 5.8 days). Those in the oldest age groups reported fewer poor health days than other ages, with those aged 40–49 reporting the highest average days with poor mental (14.4 days) and physical (5.9 days) health. Poor health days varied by race/ethnicity: Black, non-Hispanic respondents reported more days of poor physical health (8.2 days) and fewer days of poor mental health (3.7 days), while Hispanic respondents reported more poor mental health days (15.3 days) and Asian, non-Hispanic respondents reported more days of activity being impacted by poor health (8.3 days). Few differences were noted in the number of poor mental or physical health days by household size, public health sector, or education; though respondents living alone or with a partner reported more days where their activities were impacted by poor health (8.5 and 7.0 days, respectively) compared to those with larger households (3 household members: 5.5 days; 4+ household members: 5.3 days). The number of days with poor mental or physical health was slightly greater for those with the most professional experience; however, this group reported the fewest days where this poor health impacted their activities (4.7 days), while those with the least experience reported the most days of impacted activities (9.2 days).

### 3.2. Burnout of Public Health Workers

Burnout was more prevalent among some groups of respondents (Table 2). Using the standard scale (i.e., one or more symptoms of burnout), we found risk factors for burnout including years of experience and public health sector. The prevalence of burnout was 90% greater among those with 1–4 years of experience (PR = 1.90; 95% CI = 1.08–3.36) and 5–9 years of experience (PR = 1.89; 95% CI = 1.07–3.34) compared to those with <1 year of experience. Burnout was 31% more prevalent in academic respondents (PR = 1.31; 95% CI = 1.08–1.58) compared to those in public health practice.

Due to the high prevalence of burnout in our sample (66.2%), we also assessed risk factors for a high level of burnout (i.e., multiple symptoms of burnout that will not go away or need intervention). Age and years of experience were associated with high levels of burnout. Individuals aged 40–49 were 2.3 times (95% CI = 1.2–4.4) as likely to report high levels of burnout compared to those 18–29 years; and those with 10–14 years of experience were 4.3 times (95% CI = 1.1–17.2) as likely to report high burnout vs. those with <1 year of experience. No other statistically significant risk factors for burnout were identified.

Although sample sizes were small and PRs were not statistically significant, minority race appeared to be somewhat protective for burnout (Black, non-Hispanic: PR = 0.5 (0.2–1.5); Hispanic: PR = 0.7 (0.5–1.1). Having a larger household (Houseline size = 3: PR = 0.8 (0.6–1.1); Household size = 4; PR = 0.8 (0.6–1.1)) was similarly non-significantly protective against some level of burnout.

### 3.3. Impact of COVID on Career Trajectory

**Figure 1:**
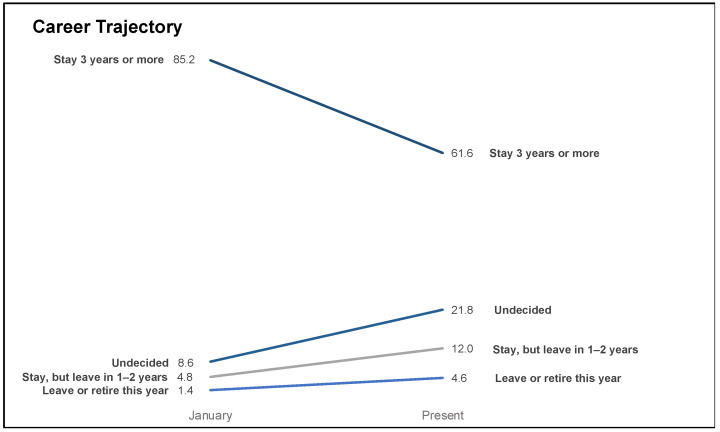
Career trajectories pre-pandemic to September 2020—United States, 23 August–11 September 2020.

The COVID-19 response has changed the career trajectory of many working in public health, with a larger share of respondents planning to leave or retire in 2020 (1.4% vs. 4.6%; *p* = 0.03) or leave or retire in 1 to 2 years (4.8% vs. 12.0%; *p* < 0.01) in September 2020 compared to their reported plans from January 2020. A decline was seen in respondents planning to stay 3 or more years pre-pandemic vs. currently (85.2% vs. 61.6%, respectively; *p* < 0.01). More respondents were undecided on future career plans in September 2020 compared to January 2020 (21.8% vs. 8.6%; *p* < 0.01) (Figure 1).

## 4. Discussion

Among public health workers surveyed, symptoms of anxiety, depression, burnout, and poor physical health were common, occuring at similar or higher levels as those reported in frontline health care workers during COVID-19 [4,5,6,7,8,9,10,11,12,13,14]. In our sample of public health professionals, 41.0% reported symptoms of anxiety and 29.1% reported symptoms of depression, while a recent meta-analysis examining these conditions among health care workers during the COVID-19 pandemic reported pooled prevalence estimates of 23.2% for anxiety and 22.8% for depression. The prevalence of poor mental health days reported by our respondents—41.4% reported poor mental health in at least 14 of the last 30 days—was also higher than the 13.8% reported in a national-level assessment of poor mental health days [25]. In prior studies, factors associated with depression among health care workers involved in epidemic or pandemic responses including being older, being married/partnered [9], and higher education [27]. Our findings were inconsistent with these findings. In our study, older age groups had lower prevalence of depressive symptoms than the youngest age group, and those with doctoral degrees also reported less symptoms of depression. We did not find being married or in a partnership was associated with lower prevalence of depressive symptoms.

Our study also found higher levels of burnout (66.2%) in public health workers than in studies of similar size examining frontline health care workers during pandemic responses. For example, in a study of Italian health care professionals during the current response, 37.0% reported high levels of emotional exhaustion [11]. With high levels of burnout, long-term consequences are also a concern. In a study of health care workers during the SARS outbreak, 30.4% of respondents showed symptoms of burnout up to 2 years after the response [9]. Therefore, as the pandemic continues into a second year, it is possible that symptoms of burnout will increase and be long lasting among the U.S. public health workforce.

An additional contributor to burnout in the U.S. public health workforce may be leadership turnover, which is disruptive to organizations during routine times and especially important during a crisis [28]. In 2020, a growing number of state and local health officials resigned or were removed from their positions due to the politicization of public health and public challenges to the implementation of control measures and the use of public health emergency powers [28]. Professional abuse and personal threats, as well as the fear of these types of attacks, have likely increased the potential for anxiety, depression, and burnout among those working in public health [29].

Among public health workers, 66.2% reported symptoms of burnout, much higher than levels identified in frontline health care workers [11,24]. Job-related burnout is likely being exacerbated by other aspects of the pandemic, particularly for females who serve as the primary caregivers for children and other family members [30]. Public health staff working in academic settings may have also been asked to take on more professional responsibility during the pandemic—learning new teaching modalities and assisting college and university administration in developing COVID-19 emergency response plans and providing pandemic response support. In this study, more experienced public health workers in both practice and academia, who are perhaps being asked to take on more roles in more functional areas, were more likely to experience extreme levels of burnout.

Comparing the career plans reported retrospectively for January 2020 and for September 2020, the pandemic response appears to be impacting career trajectories of those working in public health, most likely due to the physical and mental health effects and levels of burnout. Exhaustion, low self-efficacy, and stress have all been shown to be contributors to burnout and workforce turnover and were all present in the current sample [31,32] Prior to the pandemic, the U.S. public health workforce was already functioning at reduced capacity due to budget reductions and layoffs over the past decade [19,20]. Like other state and local government services, public health was already facing an exodus of experienced workers due to the aging workforce and their pending retirement [15,21]. Existing workforce and funding challenges, along with the stress and burnout associated with a prolonged pandemic response, will have long-lasting negative effects on public health practice and must be addressed to ensure the continued delivery of essential public health services as well as preparedness for future public health emergencies. Broad and long-lasting funding and workforce development, not just support for COVID-19 surge capacity, will be needed to ensure a robust public health system in the future [32].

This cross-sectional study has several important limitations. Compared to 2020 estimates of the demographics of the U.S. governmental public health workforce, female, White, and under 40 respondents were overrepresented in our sample, limiting the generalizability of the findings to the overall public health workforce in the U.S. due to potential sampling error [15,33]. The sample size is small; however, it is comparable to similar studies of burnout among frontline health care workers that also utilized convenience sampling. Self-selection bias is possible if respondents were motivated to complete the survey due to their high levels of mental and physical stress and burnout. However, given the extent and duration of the COVID-19 pandemic response in the U.S., and the consistency of findings across other health care professionals and public health workers in China [34] we believe that there are high levels of burnout among public health workers in general. Additionally, since the survey was shared through professional organizations and networks, self-selection bias may have resulted in public health workers who were experiencing the most burnout being more likely to respond. Career trajectory data may overestimate the change in respondent intentions between January 2020 and the time of the response since data on January 2020 intentions were collected retrospectively at the same time as the data on current trajectory. Data represent the state of the U.S. public health workforce during a three-week period approximately six months into the pandemic response, and may not reflect the mental or physical health or level of burnout at the peak of the response in their jurisdiction. However, this survey serves as a starting point for future analyses exploring the burden of a long-term response on public health workers and captures a snapshot of stress on public health workers approximately 6 months into the COVID-19 response as the pandemic response continues.

## 5. Conclusions

The public health pandemic response has been lengthy and could continue for years [35], as workers conduct surveillance, contact trace, and eventually monitor vaccine distribution. This all-hands-on-deck approach has challenged the U.S. public health workforce who must defer other public health priorities amidst an incredibly stressful and increasingly politically polarized environment. U.S. public health workforce capacity was strained pre-pandemic; while new graduates could potentially fill gaps, uncompetitive pay and limited opportunities for promotion remain challenges [21,36,37]. Investing in a robust public health workforce is critical for the response to COVID-19 and the long-term sustainability of public health preparedness and response.

## Figures and Tables

**Table 1:** Respondent characteristics and prevalence of mental and physical health outcomes related to the COVID-19 response—United States, 23 August–11 September 2020.

Mental Health and Physical Health Outcomes (%)								
Characteristic	Total *n* (%)	Anxiety Disorder	Depressive Disorder	Anxiety or Depressive Disorder	Burnout	Pool Physical Health Days (Mean)	Poor Mental Health Days (Mean)	Days Poor Mental or Physical Health Kept from Activity (Mean)
Total	225 (100)	41.0	29.1	45.6	66.2	5.0	12.4	6.7
Gender								
Female	185 (84.8)	39.9	28.2	43.8	68.3	5.1	12.1	6.7
Male	30 (13.8)	46.7	33.3	56.7	60.0	4.8	13.7	5.8
Other ^a^	<5							
Age (years)								
18–29	46 (20.4)	50.0	34.8	58.7	73.9	4.5	14.1	7.3
30–39	90 (40.0)	39.5	29.2	41.4	66.7	5.1	11.6	8.1
40–49	45 (20.0)	51.2	30.9	57.1	72.1	5.9	14.4	6.0
50–64	38 (16.9)	27.8	24.3	33.3	56.8	4.6	11.2	3.7
65+	6 (2.7)	0.0	0.0	0.0	16.7	2.7	2.8	2.2
Race-Ethnicity								
Asian, non-Hispanic	18 (8.2)	41.2	23.5	41.2	55.5	4.6	13.2	8.3
Black, non-Hispanic	6 (2.7)	0.0	0.0	0.0	33.3	8.2	3.7	3.8
Hispanic, any race(s)	21 (9.5)	45.0	30.0	45.0	50.0	5.4	15.3	6.3
White, non-Hispanic	168 (76.4)	42.1	31.9	48.2	70.1	4.6	12.3	6.7
Other race or multiple races, non-Hispanic	7 (3.2)	42.9	14.3	42.9	71.4	7.7	11.4	3.9
Marital Status								
Now Married/In Partnership	123 (55.2)	42.0	28.9	45.8	65.6	4.8	12.0	5.5
Never Married	77 (34.5)	42.1	30.3	48.7	71.4	4.8	13.1	8.2
Other ^b^	23 (10.3)	25.0	23.8	28.6	47.6	5.9	9.8	8.3
Household Size								
1	52 (23.5)	42.0	33.3	51.0	75.0	4.3	11.8	8.5
2	80 (36.2)	45.5	31.6	48.1	68.4	4.9	13.2	7.0
3	40 (18.1)	41.0	30.8	48.7	59.0	5.6	12.2	5.5
4+	49 (22.2)	35.4	20.8	36.2	59.2	5.4	11.9	5.3
Experience (years)								
<1	21 (9.4)	42.7	28.6	47.6	38.1	4.1	12.1	9.2
1–4	58 (26.0)	42.1	24.1	47.4	72.4	4.9	12.6	7.2
5–9	58 (26.0)	40.0	37.5	45.5	71.9	4.8	12.1	6.7
10–14	34 (15.3)	40.6	26.5	45.5	67.7	5.1	11.4	7.4
15+	52 (23.3)	40.4	27.5	43.1	63.5	5.4	13.1	4.7
Public Health Sector								
Public Health Practice	176 (78.9)	42.1	29.9	46.8	65.1	5.3	12.6	6.8
Academic	27 (12.1)	42.3	23.1	46.2	85.2	3.5	12.0	5.7
Other ^c^	20 (9.0)	30.0	30.0	35.0	50.0	3.9	10.3	6.6
Education								
≤Bachelors	33 (14.8)	37.5	27.3	40.6	51.5	6.0	12.3	6.3
Masters	128 (57.4)	42.9	31.5	47.6	67.2	4.2	11.9	7.3
Doctoral	62 (27.8)	39.0	25.0	44.1	72.1	6.0	13.3	5.5

Note: COVID-19 = coronavirus disease 2019; Anxiety disorder = respondents who scored ≥10 out of 21 on the Generalized Anxiety Disorder (GAD-7) scale; Depressive disorder = respondents who scored ≥3 out of 6 on the Patient Health Questionnaire (PHQ-2) scale; Burnout = respondents who scored ≥3 out of 5 on the single-item burnout measure. ^a^ Outcomes not reported with less than five respondents. ^b^ Includes widowed, divorced, separated. ^c^ Includes clinical setting, non-academic research, non-profit setting.

**Table 2:** Risk factors for burnout related to the COVID-19 pandemic response—United States, 23 August–11 September 2020.

Characteristic/Experience	Prevalence Ratio (95% CI)	
	Some Level of Burnout	High Level of Burnout
Gender		
Female	Ref	Ref
Male	0.9 (0.6–1.2)	1.0 (0.5–1.9)
Age (years)		
18–29	Ref	Ref
30–39	0.9 (0.7–1.1)	1.3 (0.7–2.6)
40–49	1.0 (0.8–1.3)	2.3 (1.2–4.4)
50–64	0.8 (0.6–1.1)	0.7 (0.3–1.9)
65+	0.2 (0.0–1.4)	^a^
Race/Ethnicity		
White, non-Hispanic	Ref	Ref
Asian, non-Hispanic	0.8 (0.5–1.2)	0.2 (0.0–1.3)
Black, non-Hispanic	0.5 (0.2–1.5)	0.6 (0.9–3.5)
Hispanic, any race(s)	0.7 (0.5–1.1)	0.7 (0.3–1.7)
Other race or multiple races, non-Hispanic	1.0 (0.6–1.7)	0.5 (0.1–3.1)
Marital Status		
Now Married/In Partnership	Ref	Ref
Never Married	1.1 (0.9–1.3)	0.6 (0.3–1.1)
Other ^b^	0.7 (0.5–1.2)	1.2 (0.6–2.3)
Household Size		
1	Ref	Ref
2	0.9 (0.7–1.1)	1.0 (0.6–2.0)
3	0.8 (0.6–1.1)	1.1 (0.5–2.3)
4+	0.8 (0.6–1.1)	1.2 (0.6–2.4)
Experience (years)		
<1	Ref	Ref
1–4	1.9 (1.1–3.4)	2.7 (0.7–10.9)
5–9	1.9 (1.1–3.3)	2.0 (0.5–8.4)
10–14	1.8 (1.0–3.2)	4.3 (1.1–17.2)
15+	1.7 (0.9–3.0)	2.8 (0.7–11.4)
Public Health Sector		
Public Health Practice	Ref	Ref
Academic	1.3 (1.1–1.6)	1.1 (0.5–2.1)
Other ^c^	0.8 (0.5–1.2)	1.2 (0.6–2.5)
Education		
≤Bachelors	Ref	Ref
Masters	1.3 (0.9–1.9)	2.3 (0.9–5.9)
Doctoral	1.4 (1.0–2.0)	2.3 (0.8–6.3)

Note: COVID-19 = coronavirus disease 2019; CI = confidence interval; Ref = reference group; Some Level of Burnout = respondents who scored ≥3 out of 5 on the single-item burnout measure; High Level of Burnout = respondents who scored ≥4 out of 5 on the single-item burnout measure. ^a^ Insufficient data for analysis. ^b^ Includes widowed, divorced, separated. ^c^ Includes clinical setting, non-academic research, non-profit setting.

## Data Availability

The data presented in this study are available on request from the corresponding author. The data are not publicly available to protect respondent privacy.
